# Using a Markov Model and Real-World Evidence to Identify the Most Cost-Effective Cholesterol Treatment Escalation Threshold for the Secondary Prevention of Cardiovascular Disease

**DOI:** 10.1007/s40258-025-00977-6

**Published:** 2025-05-24

**Authors:** Alfredo Mariani, Syed Mohiuddin, Patrick Muller, Eleanor Samarasekera, Sharon A. Swain, Joseph Mills, Riyaz Patel, David Preiss, Eduard Shantsila, Beatrice C. Downing, Michael Lonergan, Shaun Rowark, Nicky J. Welton, Rachael Williams, David Wonderling

**Affiliations:** 1 Science, Evidence and Analytics Directorate, National Institute for Health and Care Excellence, 2 Redman Place (2nd Floor), London, E20 1JQ UK; 2 Centre for Guidelines, National Institute for Health and Care Excellence, London, UK; 3 Liverpool Heart and Chest Hospital, Liverpool, UK; 4 Barts Health NHS Trust, University College London Hospitals NHS Foundation Trust, London, UK; 5 Nuffield Department of Population Health, University of Oxford, Oxford, UK; 6 Institute of Population Health, University of Liverpool, Liverpool, UK; 7 Population Health Science, Bristol Medical School, University of Bristol, Bristol, UK; 8 Clinical Practice Research Datalink (CPRD), Safety and Surveillance Group, Medicines and Healthcare Products Regulatory Agency, London, UK

## Abstract

**Background:**

Despite the decreased risk of cardiovascular disease (CVD) with statins, there remains an unfulfilled clinical need to prevent CVD events and premature mortality through further cholesterol-modifying interventions. In people with established CVD taking a statin, lipid therapy escalation to reduce low-density lipoprotein cholesterol (LDL-C) or non-high-density lipoprotein cholesterol (non-HDL-C) levels may lower the risk of CVD hospital admissions and improve survival. However, the cost-effectiveness of different cholesterol treatment escalation thresholds is uncertain.

**Objective:**

This study aimed to identify the most cost-effective cholesterol threshold for escalating lipid therapy in people with established CVD who are taking a statin, to support the 2023 update of the NICE guideline on CVD in England.

**Methods:**

A cohort Markov model with a yearly cycle length was developed to compare the lifetime costs and quality-adjusted life years (QALYs) of various LDL-C treatment escalation thresholds (0–4.0 mmol/L), using a combination of treatment effects from an original network meta-analysis of randomised controlled trials (RCTs), real-world data for estimating baseline cholesterol levels and CVD event rates from a published meta-analysis of statin RCTs. The model used the following CVD events: ischaemic stroke; transient ischaemic attack; peripheral artery disease; myocardial infarction; unstable angina; coronary revascularisation; and mortality. The model also used evidence-based estimates of resource use and costs, and published quality of life data. Baseline LDL-C levels and CVD hospital admission rates were estimated through a bespoke analysis of the English primary care data from Clinical Practice Research Datalink (CPRD), linked to Hospital Episode Statistics Admitted Patient Care (HES) and Office for National Statistics (ONS) death registrations.

**Results:**

Data from 590,917 adult individuals (61.7% men) with CVD on a statin in primary care between 1 January 2013 and 28 February 2020 were included in the CPRD-HES-ONS analysis. The most cost-effective threshold for lipid therapy escalation was an LDL-C of 2.2 mmol/L (or equivalent non-HDL-C of 2.9 mmol/L) at NICE’s lower cost per QALY of £20,000. An LDL-C of 2.0 mmol/L (or equivalent non-HDL-C of 2.6 mmol/L) was the most cost-effective treatment escalation threshold in a significant proportion (38%) of probabilistic simulations and produced more health. At this threshold, the model predicted that 42% of people with CVD would require combination therapy with ezetimibe while 19% would require an injectable drug such as inclisiran. At NICE’s upper cost per QALY of £30,000, the most cost-effective LDL-C treatment escalation threshold was 1.7 mmol/L (or equivalent non-HDL-C of 2.2 mmol/L).

**Conclusions:**

The results demonstrate the importance of establishing evidence of cost-effectiveness for cholesterol treatment escalation thresholds. The study’s findings support the updated NICE guideline recommending a threshold of 2.0 mmol/L LDL-C (or equivalent non-HDL-C of 2.6 mmol/L) for secondary prevention of CVD.

**Supplementary Information:**

The online version contains supplementary material available at 10.1007/s40258-025-00977-6.

## Key Points for Decision Markers

**Table:** 

This study used a novel approach to incorporate routinely collected real-world data to capture population heterogeneity within the structure of a pragmatic Markov model, as opposed to a more complex, time- and data-intensive discrete event simulation.
Baseline cholesterol levels were estimated using a bespoke analysis of primary care data linked to secondary care and death registration records.
An LDL-C treatment escalation threshold of 2.0 mmol/L (or equivalent non-HDL-C of 2.6 mmol/L) was recommended in NICE’s 2023 guideline update for the secondary prevention of CVD in England.

## Introduction

Cardiovascular disease (CVD) is a general term for conditions of the heart or blood vessels. Specifically, people with established atherosclerotic CVD, including coronary heart disease, peripheral artery disease, transient ischaemic attack (TIA) and ischaemic stroke, are at high risk of subsequent CVD events and mortality [1]. Although mortality from CVD has been falling in most developed countries, more individuals are now living with established CVD, which incurs a considerable cost for the public healthcare system. High levels of low-density lipoprotein cholesterol (LDL-C) are associated with an increased risk of CVD events and death. Consequently, treatment aimed at lowering LDL-C levels plays a crucial role in reducing the risk of CVD events and associated secondary hospital admissions [2]. In addition to health benefits, lowering LDL-C levels reduces hospitalisation costs [2].

Oral therapies (statins, ezetimibe and bempedoic acid) and injectable PCSK9 inhibitors (alirocumab and evolocumab) have proven LDL-C lowering and outcome benefits and are approved as lipid-lowering therapies in the UK. Inclisiran is also a potent LDL-C lowering agent, but the use of these agents is variable [3], with about one-fifth of adults in England with CVD receiving no lipid-lowering therapy [4]. This is partly because of a lack of consensus about LDL-C thresholds for therapeutic escalation for secondary prevention of CVD. On the basis of data from randomised controlled trials (RCTs), targets between 1.4 and 1.8 mmol/L have been advocated by some specialist societies and expert consensus [5, 6]. However, as of September 2023 in England, achievement of these targets has been poor, with only 42.5% of the 55.7% adults with CVD who had a cholesterol test in the last 12 months achieving either LDL-C below 1.8 mmol/L or non-high-density lipoprotein cholesterol (non-HDL-C) below 2.5 mmol/L [4].

**Fig. 1:**
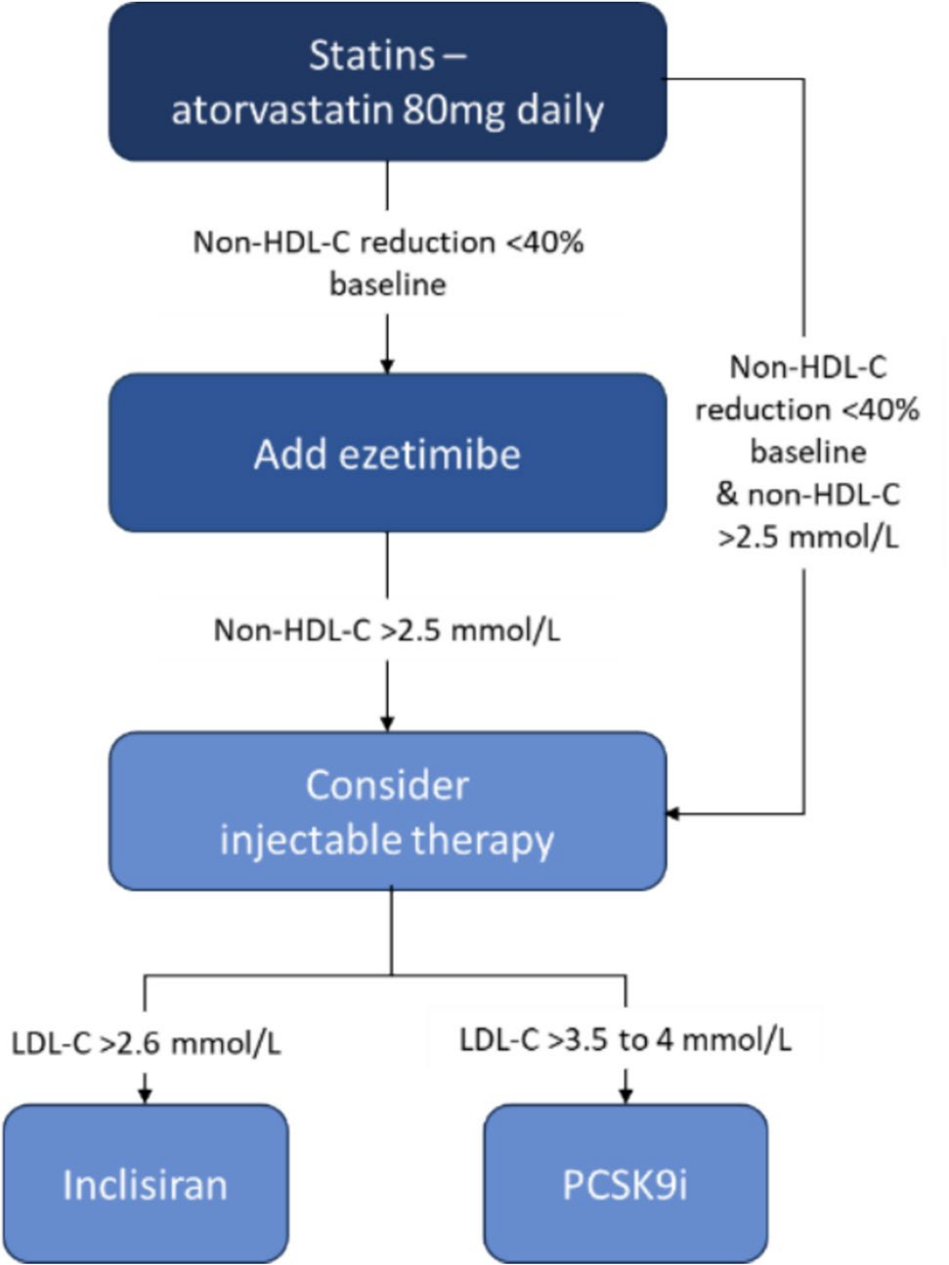
NHS Accelerated Access Collaborative pathway [13]

Historically, secondary prevention for people with CVD was informed by various guidance, including a clinical guideline for the National Health Service (NHS) in England by the National Institute for Health and Care Excellence (NICE) on ‘Cardiovascular disease: risk assessment and reduction, including lipid modification’ [7] and by five NICE technology appraisals (TA) of lipid-lowering drugs [8–12]. The pathway requires people to be initially prescribed the highest tolerated dose of statin and to be escalated to more intensive therapy, such as ezetimibe or injectable therapies, depending on a combination of relative targets and absolute thresholds, using two different measurements of atherogenic cholesterol: LDL-C and non-HDL-C (Fig. 1).

The Accelerated Access Collaborative (AAC) pathway [13] has attracted criticism for being difficult to implement and confusing. Firstly, baseline non-HDL-C values are not consistently recorded prior to initiating a statin therapy, making it challenging for a general practitioner (GP) to evaluate whether the patient has achieved the desired 40% reduction. Secondly, the sequence includes both LDL-C and non-HDL-C measurements, which are not always reported in a lipid profile test. Therefore, an update to the NICE guideline [7] was commissioned to identify the most cost-effective cholesterol threshold for lipid therapy escalation in England. Subsequently, a de novo health economic model was developed using a combination of treatment effects from an original network meta-analysis (NMA) of clinical trials, real-world data for estimating baseline cholesterol levels and CVD event rates, and a published meta-analysis of statin RCTs linking the absolute reduction in cholesterol to reduction in cardiovascular risk.

Treatment thresholds provide a level above which to start treatment, whereas treatment targets indicate the outcome to be achieved from a treatment. There is currently no evidence in support of a limit below which cardiovascular benefit of reducing LDL-C is lost [14]. However, absolute reductions in LDL-C, which determine clinical efficacy, are smaller at lower baseline LDL-C levels. Therefore, it is important that combination LDL-C treatment is initiated in people for whom it is cost-effective. The objective of this study was to determine the most cost-effective cholesterol threshold for escalating lipid therapy in people with established CVD who are taking a statin.

## Methods

### Model Structure

A cohort Markov model with a yearly cycle length was developed in Microsoft Excel to compare the costs and quality-adjusted life years (QALYs) for various cholesterol treatment escalation thresholds from the perspective of the NHS and personal social services in England. Costs and QALYs were calculated over a lifetime horizon and discounted at a rate of 3.5% per year. A range of LDL-C (0–4.0 mmol/L) and non-HDL-C (0.5–4.5 mmol/L) thresholds (divided by a step of 0.1 mmol/L) were compared with each other to identify the most cost-effective one. The main purpose of the analysis was not to alter the pharmacological pathway elicited in Fig. 1, but to identify the most cost-effective cholesterol threshold above which people would be escalated to the next treatment. However, the model also has characteristics of a target model, in that people were given a less effective (more cost-effective) medicine, if that got them just below the threshold.

**Fig. 2:**
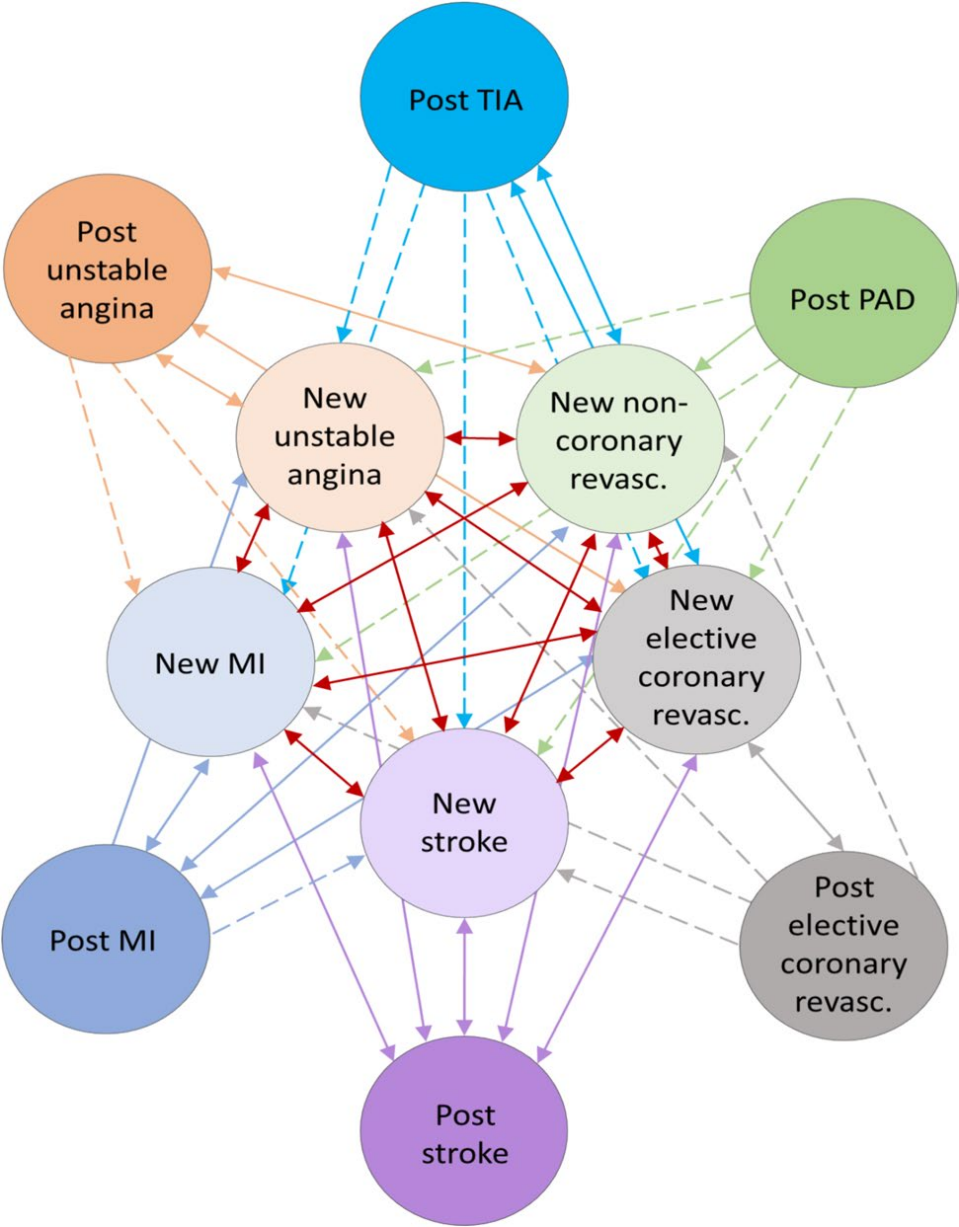
Schematic diagram of the Markov model. The dotted lines represent one-way transitions, while the solid lines represent two-way transitions. It is not shown in the diagram, but people can also remain in their current state or transition to the ‘dead’ state at the end of each cycle. MI, myocardial infarction; PAD, peripheral artery disease; TIA, transient ischaemic attack

The model structure is presented in Fig. 2, where people started in the post-event CVD states, with the starting proportions in each of these states defined by the baseline CVD event distribution. The model structure was informed by the clinical evidence collected for NICE guideline on cardiovascular disease (NG238) and the NICE guideline committee, comprising CVD specialists, generalists and patient members. The model structure is also similar to that of the statins model developed for a previous update of this NICE guideline [15]. People could transition from any alive state to any new acute CVD event state since people are always at risk of experiencing a new CVD event throughout their lifetime. If the new event was more severe (in terms of net health lost) then people would transition to the post-event state for the new event. However, if the new event was less severe, then the model moved them back to the post-event state associated with the earlier event. Although not shown in the model diagram, some people move to the dead state from each of the other states based on mortality transition probabilities specific to the state, median age and sex.

### Baseline Characteristics (CPRD-HES)

Baseline LDL-C levels were estimated using English data from Clinical Practice Research Datalink Aurum (CPRD), a large contemporary database of general practice medical record across the UK, linked to Hospital Episode Statistics Admitted Patient Care (HES APC), which records hospital admissions in England, and also Office for National Statistics (ONS) death registrations. In total, routinely collected data from 590,917 people with prior CVD and prescribed a statin in primary care (38% women) between January 2013 and February 2020 were used in the analysis.

Patients were included from the date of the first statin prescription they received after their first CVD event (index CVD). February 2020 was chosen as a cut-off as mortality and hospital admission rates during the height of the COVID-19 pandemic would not be generalisable to the present day. Patients were excluded if they received a prescription for ezetimibe, bempedoic acid, or an injectable cholesterol lowering drug (alirocumab, evolocumab, inclisiran) before their first statin. The study population was created by CPRD staff, who had full access to the underlying databases. Annual rates of CVD and mortality were calculated as the number of events per person-year. This was determined by dividing the total number of events occurring in a year by the total person-years at risk prior to any censoring. Generally, censoring in the analysis occurred when the patient died, the date cut-off was reached, the patient de-registered from the practice, their statin therapy was discontinued, or their therapy was escalated to include an additional lipid-lowering drug. However, for mortality in the 12 months after an event, censoring only took place at the point of death or the end of the follow-up period. This was to ensure mortality associated with an acute event was not under-estimated due to treatment switching. Cholesterol measurements available in the dataset were used to establish a baseline cholesterol distribution in the population and to estimate changes in cholesterol over time. Further information on the code lists used to identify CVD episodes and mortality, as well as the event rates, are provided in Supplementary Material.

**Fig. 3:**
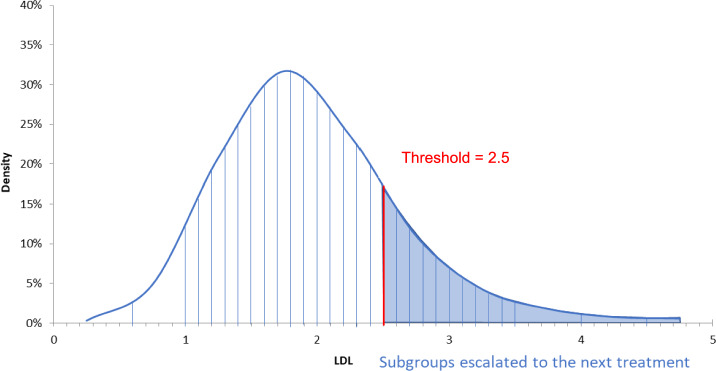
Subgroups by LDL-C with an example 2.5 mmol/L threshold

An LDL-C distribution curve was constructed for each gender, and each curve was divided into 30 subgroups to capture a wide range of different cholesterol levels (Fig. 3). These subgroups were used to determine the risk of subsequent CVD events across the population of interest and were separately simulated by the Markov model since each had its own sex, baseline (median) age and baseline LDL-C level. The model systematically applied all potential thresholds from 0 to 4 mmol/L. Everyone above the threshold received the first line of escalation treatment, ezetimibe, and if still above the threshold, the next and last line of treatment, namely injectable therapy with either inclisiran or PCSK9 inhibitors. Change in LDL-C over time was incorporated into the model and informed from the CPRD-HES analysis, such that some subgroups that were below the threshold in the first cycle of the model advanced above it later and subsequently had their lipid therapy escalated at that time.

Cholesterol was measured annually as part of routine monitoring as well as 3 months after initiating a new treatment. At the beginning of each cycle, people whose LDL-C was above the threshold received the next step of the escalation, to reduce their cholesterol (and consequently their risk of another CVD event and mortality). The model was developed such that cholesterol treatment escalation could take place once or even twice within a single cycle if the mean LDL-C in a specific subgroup remains above the threshold after the first escalation treatment. In this scenario, treatment effects of multiple treatments are applied within the same cycle.

At each cycle, people were at risk of a new CVD admission or death. CPRD data were used to estimate admission rates for each type of the CVD event, stratified by age and gender. These were then used to calculate the transition probabilities for each disease included in the Markov model. A new acute CVD state and a post-event CVD state were defined for each event type. In the new acute CVD states, mortality rates (by age, sex and admission type in the last 12 months) were taken from the CPRD-HES-ONS data. In the post-CVD states, the model applied the mortality rates from the CPRD-HES-ONS observed in those with no acute CVD event in the last 12 months (Supplementary Material).

### Rates by Cholesterol Level

Different transition probabilities were estimated for each subgroup depending on their LDL-C level. A well-recognised way of estimating change in CVD risks associated with changes in LDL-C is recommended in a consensus statement by the European Atherosclerosis Society (EAS) [16] and it is based on analysis by the Cholesterol Treatment Trialists’ (CTT) collaboration [2]. This approach was undertaken by different analyses on lipid-modification treatment, including the NICE TA on inclisiran [11] and several studies [17] on lipid-lowering therapies. The CTT collaboration has conducted various meta-analyses of statin trials; it has shown that lowering LDL-C by 1 mmol/L is associated with a proportional reduction in the rate of major CVD events of 22%. The CVD event-specific relative risk reductions from CTT are shown in Table 1.

**Table 1:** Relative effect on cardiovascular events and mortality per 1 mmol/L reduction in LDL-C

Event	Application in model base-case	Relative risk (95% confidence interval)	Source
Major cardiovascular event	Non-coronary revascularisation	0.78 (0.76 to 0.80)	CTT collaboration 2010 [2]
Any coronary revascularisation	Elective coronary revascularisation	0.74 (0.71 to 0.79)	Weighted average using CTT collaboration 2010 [2]
Ischaemic stroke	Ischaemic stroke	0.78 (0.69 to 0.80)	Weighted average using CTT collaboration 2010 [2]
Myocardial infarction	Myocardial infarction	0.73 (0.67 to 0.80)	Weighted average using CTT collaboration 2010 [2]
Coronary heart disease death	CVD death (sensitivity analysis only)	0.80 (0.74 to 0.87)	CTT collaboration 2010 [2]
All-cause mortality	All deaths	0.90 (0.87 to 0.93)	CTT collaboration 2012 [18]

The EAS [16] proposed the following equation to calculate the relative risk reduction of CVD events:

1 $$Risk reduction=1-{RR}^{LDL*Tx}$$

where *RR* is the relative risk reduction, like those in Table 1, LDL is baseline LDL-C and *Tx* is the treatment effect expressed as a percentage reduction in mmol/L. Based on the above equation, the following equations were defined:

2 $${R}_{x}={R}_{0} \times {RR}^{{LDL}_{x}}$$

3 $${R}_{\text{all}} ={R}_{0}\times ({p1.RR}^{c1}+{p2.RR}^{c2}\dots {p16.RR}^{c16})$$

where ${R}_{x}$ is the cardiovascular risk of subgroup *x*, ${R}_{0}$ is the hypothetical cardiovascular risk the subgroup would incur if their LDL-C was reduced to 0, *RR* is the relative risk reduction per 1 unit reduction in mmol/L from the CTT collaboration study, ${LDL}_{x}$ is the actual LDL-C level of subgroup *x,* ${R}_{\text{all}}$ is the overall event rate for age-sex subgroup, *p1* is the proportion of age-sex cohort that are in cholesterol subgroup 1 and *c1* is the mean cholesterol in cholesterol subgroup 1 of age-sex cohort. Equation 2 followed the same approach as Eq. 1 but allowed estimation of LDL-C-specific risk across the whole distribution of LDL-C. Equation 3 shows how the overall risk observed in the CPRD-HES data could be expressed as the weighted average of the risks occurring in each subgroup. ${R}_{0}$ is calculated by populating and rearranging Eq. 3 and then is fed into Eq. 2 to estimate the event rate for each subgroup. This process was repeated for each outcome in Table 2.

**Table 2:** Relative reduction in LDL-C and non-HDL-C on background therapy of statins from network meta-analysis (random-effects)

	LDL-C* (95% credible interval)	Non-HDL-C* (95% credible interval)
Ezetimibe versus placebo	−17.8% (−23.7% to −11.9%)	−20.0% (−33.0% to −6.9%)
Inclisiran versus placebo	−51.3% (−61.9% to −40.5%)	−45.1% (−58.6% to −31.0%)
PCSK9 inhibitors versus placebo	−55.0% (−60.3% to −49.4%)	−47.0% (−54.3% to −39.4%)

*NMA of LDL-C included 19 RCTs, while NMA of non-HDL-C included 14 RCTs

**Fig. 4:**
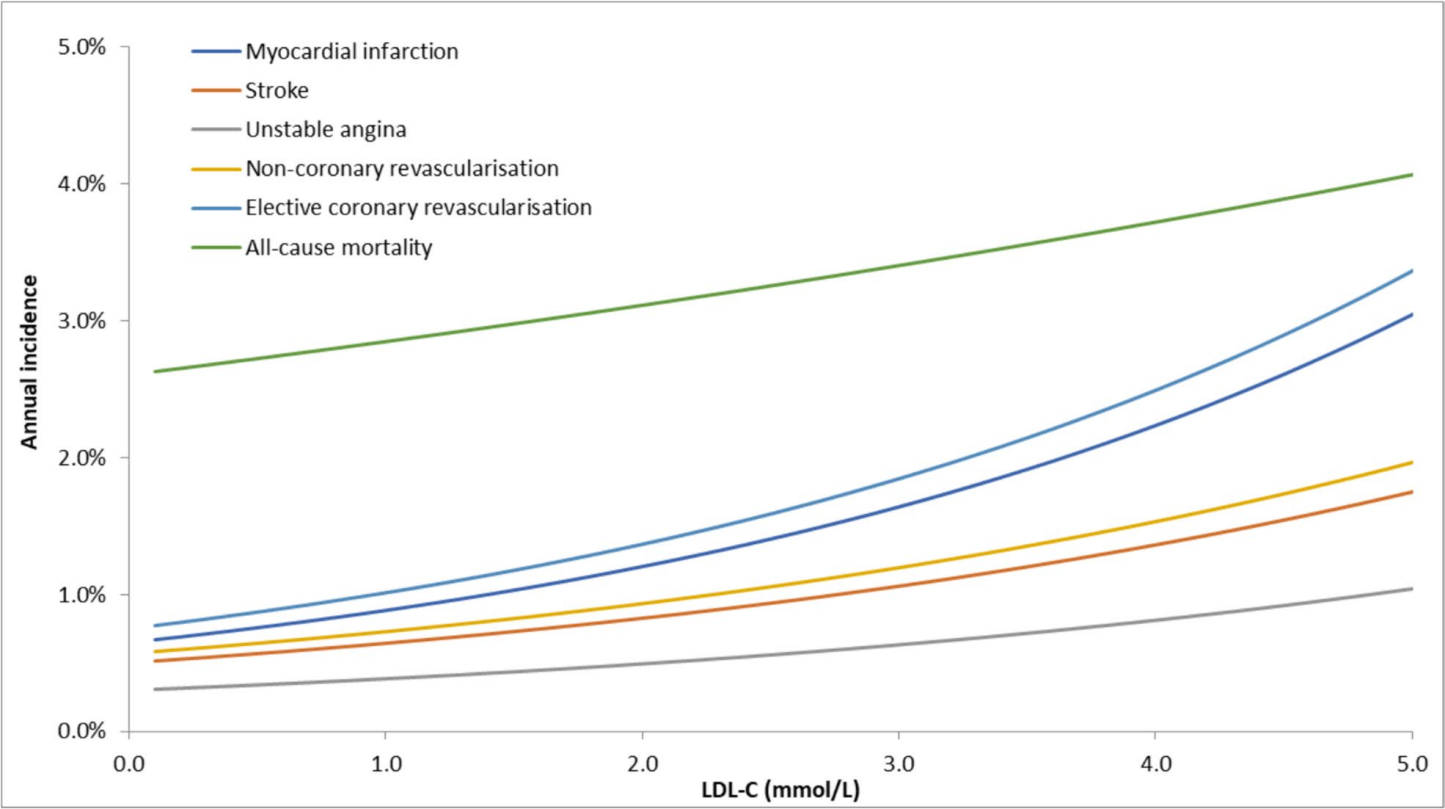
Annual event rates for a 70-year-old man by LDL-C level

Figure 4 illustrates an example featuring a 70-year-old man. Events that were less influenced by cholesterol, such as all-cause mortality, exhibit a slower growth as LDL-C level increased. Conversely, events strongly associated with cholesterol, such as myocardial infarction (MI) or coronary revascularisation, showed a significantly steeper growth rate with increasing LDL-C level. This approach was used in the model to dynamically estimate how event risks vary as cholesterol changes.

The mortality effect was calibrated to avoid potential overestimation in mortality reduction caused by the simultaneous application of a direct treatment effect on mortality and the indirect effect mediated by reduction of the other CVD events. To achieve this, an adjustment factor was applied to the mortality treatment effect to ensure that the overall mortality reduction predicted by the model was identical to that observed in the CTT study.

### Treatment Effects

The treatment effect of any medicine included in the pathway was expressed in terms of percentage LDL-C reductions that were applied to the baseline LDL-C level of any subgroup above the treatment escalation threshold. A systematic review and NMA were conducted [19] to estimate the treatment effect of all escalation therapies in addition to continued background statin therapy compared with placebo (Table 2). Owing to the heterogeneity observed, a random-effects model was chosen for the NMA to reflect between-study variation.

The systematic review of RCTs found no evidence of significant treatment-related adverse events [19]. Therefore, none were included in the model. There were some injection site-related adverse events noted for injectable therapies, but these were minor and transient and so would not impact on cost or quality of life.

### Utilities

Age- and sex-specific quality of life scores (‘utilities’) were used in the model. They were derived from Health Survey for England (HSE) data as reported in a publication by the NICE Decision Support Unit [20]. HSE data from 2017 [21] were used to estimate the impact on quality of life of the CVD events in their acute and post phases. Utility multipliers were calculated as the mean EQ-5D utility score of people who had experienced a specific CVD event divided by the mean EQ-5D utility score of the whole sample, adjusted for age and gender (Table 3).

**Table 3:** Utility multipliers used in the model

Cardiovascular event	Acute state mean (SD)	Post state mean (SD)	Rank (1 = worst; 5 = least worst)
Stroke	0.756 (0.064)	0.816 (0.013)	1
Unstable angina	0.682 (0.021)	0.878 (0.011)	3
Myocardial infarction	0.839 (0.054)	0.847 (0.010)	2
Peripheral artery disease	–	0.927 (0.016)	5
Elective coronary revascularisation	–	0.889 (0.028)	4

### Costs

Medication dosages were obtained from the British National Formula (BNF) [22], and NHS Drug Tariffs [23] were used for drug prices when publicly available. Prices that were subject to a commercial access agreement (inclisiran) or patient access schemes (PCSK9 inhibitors) between manufacturers and the NHS are commercial-in-confidence and, although used in the analysis, cannot be reported in this paper. It was assumed that people undergo an annual lipid test for routine monitoring and that an escalation of therapy would require a GP appointment, except for the two PCSK9 inhibitors that would require a hospital outpatient visit (Table 4).

**Table 4:** Unit costs used in the model

Resource	Unit cost	Source
Statin (atorvastatin)	£1.40 for 28 tablets	BNF [22] and NHS Drug Tariff [23]
Ezetimibe	£1.47 per 28 tablets	BNF [22] and NHS Drug Tariff [23]
Inclisiran	In confidence	Commercial access agreement
PCSK9 inhibitors	In confidence	Patient access schemes
Lipid test including phlebotomy	£6	NHS reference costs 2019/2020 [24]
Nurse visit (including qualification costs)	£11	PSSRU 2020/2021 [25]
GP appointment (including qualification costs)	£38	PSSRU 2020/2021 [25]
Outpatient visit	£138	NHS reference costs 2019/2020 [24] WF01A

The annual healthcare costs associated with different CVD events were obtained from peer-reviewed literature and were stratified into the year of the event (event year) and following years (post-event years) and were inflated to 2022/2023 when necessary. Costs for ischaemic stroke admissions, MI admissions, elective coronary revascularisation admissions and cardiovascular deaths were obtained from a recently published study that used the UK Biobank dataset [26] to estimate the impact of incident CVD events on primary care (including primary care consultation, diagnostic and monitoring tests, and prescription medicines) and hospital care costs over a 10-year period from 2006 to 2016. There were interaction terms for when multiple events occurred in the same year, such as MI admissions with vascular death, stroke with vascular death and MI admissions with elective coronary revascularisation admissions. The model captured these costs using the coefficients of relevant interaction terms. Likewise, costs were adjusted for mean age and gender of each subgroup using the corresponding interaction terms

Apart from healthcare cost, social care costs (e.g. costs of care home, home help, meals on wheels and social service day centre visits) were also included for patients suffering a stroke, and it was assumed that 50% of this cost was covered by out-of-pocket payments from patients. This is in line with assumptions made in a previous NICE guideline NG208 [27]. Table 5 shows the estimated costs of stroke, MI and elective coronary revascularisation for each gender and age group.

**Table 5:** Acute cost (in the event year) of stroke, myocardial infarction admissions and elective coronary revascularisation admissions by age and gender

Age	Stroke (including cost of social care)		Myocardial infarction admissions		Elective coronary revascularisation admissions	
	Male	Female	Male	Female	Male	Female
50–54	£11,610	£11,799	£7706	£8067	£7667	£7854
55–59	£11,636	£12,076	£7869	£8314	£7780	£7955
60–64	£11,952	£12,438	£8150	£8474	£7902	£8079
65–69	£12,325	£12,774	£8464	£8854	£8027	£8204
70–74	£12,545	£12,830	£8856	£9053	£8174	£8340
75–79	£12,913	£13,109	£9244	£9308	£8409	£8509
80–84	£13,234	£13,467	£9740	£10,123	£8619	£8738
85–90	£13,573	£13,873	£10,321	£10,447	£8928	£9064
> 90	£14,216	£14,632	£11,278	£11,378	£9392	£9676

Costs calculated using the coefficients obtained from Zhou et al. [26]

The costs of the remaining events and their sources are provided in Table 6.

**Table 6:** Costs of other CVD episodes

Resource	Unit cost	Source
Transient ischaemic attack (TIA) episodes	Acute cost: £3196 Post-event cost: £327	Danese et al. [28]
Unstable angina pectoris admissions	Acute cost: £8835 Post-event cost: £428	Acute cost: NHS reference cost 2019/20 [24] Post-event cost: Walker et al. [29]
Non-coronary revascularisation admissions	£2720	Zhou et al. [26]
Vascular deaths	Acute cost: £3196 Post-event cost: £327	Danese et al. [28]

### Scenario Analysis

Several sensitivity analyses were conducted to assess the robustness of the results against the various assumptions used in the base-case analysis (Table 7). These included using different parameters for relative risk reductions, treatment adherence, quality of life, and inclisiran price; using a CVD mortality effect instead of an all-cause mortality treatment effect; including PCSK9 inhibitors as a further or alternative escalation treatment to inclisiran; expanding the population to include people who are intolerant to statin; and excluding some CVD events from the analysis.

**Table 7:** Assumptions used in the base-case scenario and scenario analyses

Feature	Scenario	Description
Cost-effectiveness threshold	£20,000 per QALY	NICE’s lower threshold
	£15,000 per QALY	Opportunity cost estimate used by the Department of Health and Social Care
	£30,000 per QALY	NICE’s upper threshold
Relative risk reduction	Event-specific relative reduction*	Used a different event-specific treatment effect for each outcome
	Single major cardiovascular events (MACE) relative reduction	Used MACE treatment effect for all CVD outcomes
Effect of LDL-C reduction on mortality	LDL-C reduction affects all-cause mortality*	Corresponding relative risk from CTT was applied to all-cause mortality
	LDL-C reduction affects CVD mortality only	Corresponding relative risk from CTT was applied to CVD mortality only
PCSK9 inhibitors	Inclisiran only*	Nobody escalated to PCSK9 inhibitors. People above the threshold who were taking ezetimibe received inclisiran
	PCSK9 inhibitors only	Nobody escalated to inclisiran. People above the threshold after taking ezetimibe received a PCSK9 inhibitor
	PCSK9 inhibitors at 3.5 mmol/L	People escalated to PCSK9 inhibitors if their LDL-C was above 3.5 mmol/L
Population	People on any statin*	Analysis on people on any statin
	People on atorvastatin 80mg	Used the age/sex/LDL-C distribution for the subgroup of people on atorvastatin 80 mg
	People on any statin and people who are statin intolerant	The base-case population ran through the model then the statin intolerant population ran through the model using an alternative treatment sequence. Both populations were treated to the same threshold and weighted average results were calculated
Angina	Include unstable angina*	Included unstable angina admissions
	Exclude unstable angina	Excluded angina from the model
TIA	Include TIA*	Included TIAs (costs only)
	Exclude TIA	Excluded TIA costs from the analysis
Quality of life weights	Health Survey for England 2017*	Applied the quality-of-life multipliers calculated from the HSE 2017
	Old version of statins model	Applied the quality-of-life multipliers used in the 2014 version of the NICE guideline (NG181)
	Inclisiran TA	Applied the quality-of-life multipliers used in the inclisiran TA
Adherence to ezetimibe	100% adherence*	Assumed a 100% adherence to ezetimibe
	80% adherence	Assumed an 80% adherence to ezetimibe (that is for 20% of patients with no cost of ezetimibe and no benefit either)
	50% adherence	Assumed a 50% adherence to ezetimibe (that is for 50% of patients with no cost of ezetimibe and no benefit either)
Adherence to injectable therapies	100% adherence*	Assumed a 100% adherence to injectable therapies
	80% adherence	Assumed an 80% adherence to injectable therapies (that is for 20% of patients with no cost and no benefit either)
	50% adherence	Assumed a 50% adherence to injectable therapies (that is for 50% of patients with no cost of ezetimibe and no benefit either)
Inclisiran price	Invoice price*	Used the invoice price of inclisiran that the NHS is currently charged for
	Volume discounted price	Used volume discounted price that is applicable once specific patient volumes are achieved
Escalation to inclisiran	One GP attendance*	Assumed that one GP attendance is sufficient to be prescribed inclisiran
	Two GP attendances and one nurse attendance	Added an extra GP attendance and a nurse-led attendance
Ezetimibe prescription fee	No prescription fee*	The cost of ezetimibe does not include the prescription fee paid to the pharmacist
	Including prescription fee	The cost of ezetimibe includes the prescription fee paid to the pharmacist
Cholesterol changes over time	Gradual constant lifetime increase adjusted for gender*	LDL-C changes over time using a gender-specific rate
	LDL-C regresses to the mean over 3 years	LDL-C change for three cycles using a gender, age and baseline cholesterol-adjusted model

*Base-case assumption

In the base-case scenario, LDL-C was allowed to increase every cycle using the estimation from CPRD. However, it was noted that LDL-C change may be subject to regression to the mean. This implies that the benefits of escalation may be overestimated for people at a high baseline cholesterol level but underestimated for people at a low baseline LDL-C level. Therefore, a further statistical model was specified that calculated change in LDL-C from the same CPRD dataset using covariates for initial LDL-C, age, gender and interactions between those terms. This was used in a scenario analysis where the LDL-C was allowed to vary over the first three cycles (reflecting mean follow-up time in the sample) separately for each LDL-C or gender subgroup – increasing in subgroups with lower baseline LDL-C but decreasing in those with higher baseline LDL-C levels.

The base-case LDL-C analyses were run probabilistically for 10,000 simulations to take account of the uncertainty around input parameters. Probability distributions were defined for most model input parameters and the type of distribution used was based on the properties of data of that type. Gamma distributions were used for rates, costs and disutilities, beta distributions for probabilities and lognormal distributions for treatment effects. Analyses were then repeated using non-HDL-C in place of LDL-C (Supplementary Material).

## Results

### Base-Case Scenario

Table 8 shows the fully incremental analysis on a range of LDL-C treatment escalation thresholds between 0.8 and 4 mmol/L. Lower thresholds resulted in a higher percentage of people treated with ezetimibe or inclisiran, lower LDL-C and higher mean QALYs per patient. The most cost-effective threshold for lipid therapy escalation was found to be 2.2 mmol/L at a cost per QALY threshold of £20,000. Results stratified by sex are shown in Tables S7 and S8 of the Supplementary Material.

**Table 8:** Proportion of people on medication, mean QALYs and mean LDL-C at different thresholds – deterministic results

Threshold LDL-C (mmol/L)	Mean QALYs	% of people on ezetimibe at 1 year	% of people on inclisiran at 1 year	Mean LDL-C at 1 year (mmol/L)
4.0	5.959	1.5%	0.7%	1.91
3.1	5.973	5.9%	1.5%	1.87
2.7	5.994	13.2%	4.2%	1.79
2.4	6.021	22.4%	7.8%	1.70
2.2	6.046	31.2%	13.2%	1.60
2.0	6.078	42.3%	18.8%	1.50
1.8	6.115	50.6%	31.2%	1.35
1.5	6.174	73.9%	50.6%	1.12
1.2	6.225	88.7%	74.6%	0.92
0.8	6.253	99.6%	94.8%	0.79

Note: costs are not reported due to the confidentiality agreement on the price of inclisiran

As the distribution of cholesterol loosely resembles a normal distribution with most observations located at the centre of the distribution, even a slight reduction in the escalation threshold could disproportionally increase the number of people requiring treatments. This is particularly evident in Table 9, which compares the probabilistic results of three different thresholds that lie around the centre of the cholesterol distribution. Although reducing the escalation threshold from 2.2 to 2.0 mmol/L increased the proportion of people requiring inclisiran by 5%, a further reduction to 1.8 mmol/L caused an additional 13% of people to be eligible for inclisiran, with a significant impact on NHS spending. This is the reason that an LDL-C escalation threshold of 1.8 mmol/L was found to be cost-effective at £20,000 per QALY in only 3% of the simulations.

**Table 9:** Comparing thresholds – probabilistic results

Thresholds	1.8 mmol/L	2.0 mmol/L	2.2 mmol/L
Mean QALYs	6.116	6.080	6.048
Proportion of people requiring ezetimibe	50.6%	42.3%	31.2%
Proportion of people requiring inclisiran	31.2%	18.8%	13.2%
Probability cost-effective at £20,000 per QALY	3%	38%	59%

Note: costs are not reported in the any table due to the confidentiality agreement on the price of inclisiran

### Scenario Analysis

Table 10 shows the optimal treatment escalation threshold of various LDL-C analyses, mean QALYs and proportion of people on each treatment in all the scenarios tested. The results of the non-HDL-C analyses are provided in the Supplementary Material. In most scenarios, the optimal threshold remained close to 2.0–2.2 mmol/L, including a scenario where a sub-population that is intolerant to statins was added. This suggested that the optimal threshold could be broadly applied to everyone in the CVD secondary prevention population.

**Table 10:** Scenario analyses – deterministic results

	Optimal threshold LDL-C (mmol/L)	Mean QALYs	% on ezetimibe	% on inclisiran
Base-case	2.2	6.046	31.2%	13.2%
£15,000 per QALY	2.4	6.021	22.4%	7.8%
£30,000 per QALY	1.7	6.136	61.3%	32.9%
CVD mortality RR	2.7	5.986	9.8%	3.7%
Only PCSK9i	3.2	5.969	4.2%	1.5%
PCSK9i at > 3.5 mmol/L	2.2	6.047	31.2%	13.2%
Atorvastatin cholesterol distribution	2.2	7.753	20.7%	8.9%
Exclude unstable angina	2.2	6.059	31.2%	13.2%
Exclude TIA	2.2	6.046	31.2%	13.2%
Previous statin model utilities	2.2	5.800	31.2%	13.2%
Inclisiran TA Utilities	2.0	6.338	42.3%	18.8%
Ezetimibe 80% adherence	2.4	6.021	17.9%	9.8%
Ezetimibe 50% adherence	2.7	5.993	4.9%	5.9%
Ezetimibe 0% adherence	3.1	5.985	0%	5.9%
Injectables 80% adherence	2.0	6.063	42.3%	15%
Injectables 50% adherence	1.9	6.053	50.6%	11.8%
Injectables 0% adherence	0.8	6.042	99.6%	0%
Different CVD event costs	2.2	6.202	31.0%	12.9%
Volume discounted inclisiran price	1.9	6.095	50.6%	23.7%
Higher inclisiran escalation cost	2.2	6.046	31.2%	13.2%
Pharmacist fee with ezetimibe	2.2	6.046	31.2%	13.2%
Three-cycles LDL-C change adjusted for gender and baseline LDL-C	2.1	6.053	32.9%	14.0%
Statin intolerance	2.2	6.044	37.5%	15.0%

Note: costs are not reported in the table due to the confidentiality of the price of inclisiran and the PCSK9 inhibitors

## Discussion

This model-based cost-effectiveness study was conducted to inform an update to the NICE guideline on CVD [7] and to identify the most cost-effective cholesterol threshold for lipid therapy escalation in England in people with established CVD on a statin. At a cost per QALY of £20,000, 2.2 mmol/L LDL-C (or equivalent 2.9 mmol/L non-HDL-C) was the most cost-effective threshold for lipid therapy escalation in 59% of probabilistic simulations, while 2.0 mmol/L LDL-C was the most cost-effective in 38% of probabilistic simulations. The results were robust to most sensitivity analyses, but sensitive to the cost-effectiveness threshold: the optimal escalation threshold went up to 2.4 mmol/L when a lower cost per QALY threshold of £15,000 was used and went down to 1.7 mmol/L when a higher cost per QALY threshold of £30,000 was used. The addition of the statin-intolerant population did not affect the optimal value of the LDL-C escalation threshold. The CTT relative reductions were based on LDL-C and the NMA of LDL-C included more evidence than the NMA of non-HDL-C; as such, the main emphasis was around LDL-C levels.

It was not possible to validate the model results using the trial data because of differences in patient demographics and baseline cholesterol levels. However, overall, the relative reduction in major CVD events predicted by the model was very similar to that observed in the clinical trials for PCSK9 inhibitors and ezetimibe (Table 11). Inclisiran is a relatively recently approved drug and although its effect on reducing cholesterol has been adequately proven, there is a scarcity of trials showing its effects on cardiovascular events. Although recent trials such as ORION-10 and ORION-11 [30] have showed a higher relative reduction than the one predicted in the model, this outcome was exploratory in the trials and not adjudicated by an independent clinical committee.

**Table 11:** Major cardiovascular events relative risk reduction – model results compared with RCTs

	Model – entire cohort (lifetime)	RCTs (meta-analyses from evidence review – various follow-up points)
Ezetimibe	−7%	−6%
Inclisiran	−16%	−26%
PCSK9 inhibitors	−17%	−17%

The analysis employed a novel approach that combined real-world evidence on baseline cholesterol levels, treatment effects from an original NMA of RCTs and the relationship between cholesterol and CVD event rates from a published meta-analysis of statin RCTs. Data from 590,917 adults in the CPRD-HES-ONS linked dataset were used to conduct longitudinal analyses of demographics, cholesterol changes over time, CVD events and prescription episodes. A key feature of economic analyses of CVD is the heterogeneity of patients in terms of cholesterol levels, which are strong predictors of future CVD events and mortality. In the literature, this problem has been addressed by utilising individual patient simulation models [17]. In this study, heterogeneity was captured within a cohort Markov model framework by using several subgroups that differed in demographic characteristics, lipid level, and consequently, their risk of CVD events and mortality. Each subgroup functioned as a ‘super patient’ whose lifetime and CVD events were simulated, representing a cohort of individuals with similar characteristics. This approach enabled the analysis of a complex question using a relatively simple and flexible Markov model structure, as opposed to a more complex, time- and data-intensive individual patient simulation. A similar method was employed in a published model [31] that estimated the cost-effectiveness of diet and physical activity interventions.

Markov models offer a pragmatic solution within the NICE decision-making process, where transparency is essential and accessibility is necessary to enable a range of stakeholders to critically appraise the model. However, the use of a Markov model presented certain limitations, as it could not fully capture all forms of heterogeneity (e.g. age) or account for interactions between some concurrently occurring conditions (e.g. stroke following a TIA). When the goal is to identify specific population groups that benefit most from prevention policies, microsimulation models are generally the preferred approach. However, within the context of a NICE guideline – where recommendations are broad and intended for application across varied population groups – this method was deemed adequately robust.

This study used commercial-in-confidence prices of the drugs that are subject to a patient access scheme or commercial access agreement. This meant that costs and cost-effectiveness ratios needed to be omitted to avoid back-calculation of the commercial-in-confidence prices. Furthermore, since we were calculating a cost-effective threshold, some other data in the model had to be omitted to avoid back-calculation of the confidential prices, specifically the numbers and median age in each population subgroup. The price discounts are substantial, such that use of list prices in the model would have given an unrealistically high cost-effective threshold. Hence there is a trade-off between using realistic prices and transparency of the model.

Comparing the results of this study with other similar studies on lipid thresholds is challenging given the use of NHS England-specific commercial-in-confidence prices of the drugs to determine the most cost-effective cholesterol threshold. This is a common issue for models looking at treatment thresholds or targets that make comparisons with other studies particularly difficult. A Swedish study [32] evaluated the predicted impact of reducing the LDL-C of a sample of people from a Swedish national register below 1.8 mmol/L compared with current practice; however, the study considered the benefits and cost savings of cholesterol reduction only, without the costs of the therapies needed to achieve the desired reduction. A German study [33] evaluated the demand of PCSK9 inhibitors and healthcare costs associated with the LDL-C target of 1.4 mmol/L outlined by the European Society of Cardiology, and found that reaching such a target would pose significant affordability challenges for any healthcare system. This shows that setting lipid targets is very demanding for any healthcare system and should be done alongside a rigorous cost-effectiveness analysis.

This study has a number of strengths and limitations. Reductions in rates of CVD events were not applied using observed outcomes in clinical trials but estimated using an indirect cholesterol-mediated approach. This indirect approach, although apparently a limitation, addressed a potential shortcoming of clinical trials, where short follow-ups often fail to capture the true impact of lipid-lowering therapies, which typically manifest gradually over a longer period. However, the delay between treatment and effect was not captured in the model, which assumed that a reduction in LDL-C would instantly translate into a reduction in CVD risk. This, however, did not seem to introduce biases in the long-term results as observed in Table 11.

Most of the model parameters were estimated using real-world evidence from a CPRD-HES-ONS linked dataset. Although this ensured that the analysis was highly applicable to the English setting, a few limitations were observed given that the data were not created or collected for the purposes of research, particularly regarding TIA and angina admissions. TIA does not typically require hospitalisation and is mostly recorded in primary care; however, the same episode could be recorded multiple times. Angina could potentially encompass admissions for undifferentiated chest pain that would not benefit from a lipid-lowering therapy. To address this concern, both TIA and angina events were adjusted using external data and excluded in scenario analyses. All-cause and CVD-specific mortality treatment effects were available from the CTT collaboration and were applied to the corresponding mortality rates in two separate scenarios. The first scenario, with all-cause mortality, was considered more appropriate for the base-case analysis as CPRD showed that non-CVD mortality increased after a CVD event.

Escalation to a further therapy in the model was based on a single annual measurement of cholesterol above the threshold, which made any assumptions on cholesterol variation over time particularly important. The model assumed that, without changes in treatment, cholesterol increases gradually over time at a constant rate, informed by an analysis of the CPRD-HES-ONS linked dataset. To address potential biases caused by regression to the mean, a further scenario was tested, which resulted in a slightly lower optimal cholesterol threshold.

The base-case analysis of this study included a population with CVD who are taking a statin regularly, but a sensitivity analysis of a statin intolerant population found no difference in the value of the optimal single LDL-C escalation threshold. Importantly, the results cannot be generalised to people using statins for primary prevention, as their risk of suffering a CVD event would likely be much lower. Medicines were assumed to be prescribed in primary care, except for PCSK9 inhibitors (alirocumab and evolocumab), which require an outpatient appointment, and taken with 100% adherence. The scenario analysis showed that changes in adherence could potentially affect the target, but no data on real-world adherence to these drugs was available. If adherence to the new medication is low, achieving targets would become significantly more difficult, posing a challenge for secondary prevention. At the time of this analysis, inclisiran was seldom prescribed in primary care, so the burden often falls on secondary care. If this persists, the costs would be higher than those estimated in the model, and the threshold would consequently increase. This study used UK-specific cholesterol distribution, admission rates and commercial-in-confidence prices for inclisiran and PCSK9 inhibitors. As such, the most cost-effective cholesterol threshold for lipid therapy escalation presented here may not be generalised to other countries or jurisdictions. However, the principles and methods presented here could be used with country-specific inputs.

This study identified the most cost-effective cholesterol treatment escalation threshold for people with CVD from the perspective of the NHS in England. Cholesterol targets and thresholds are popular with healthcare professionals, agencies and patients. Given that some medicines are available generically while others are much more costly, it might be more efficient to use algorithms with cholesterol treatment escalation thresholds that are specific to each treatment instead. However, there might still be reasons for healthcare organisations to use algorithms with a single cholesterol threshold to improve population health. Firstly, uptake of lipid-lowering treatments has been suboptimal, and a threshold can provide an incentive for patients and clinicians to make the most of these effective treatments, especially if tied in with financial payments. The NICE treatment escalation threshold has been linked in with payments to GPs in England through the Quality Outcomes Framework. Secondly, it can be argued that there is a more equitable outcome by treating everyone with CVD to the same target rather than using medicine-specific treatment thresholds, which would lead to more treatment at the low end of the risk distribution at the expense of less intensive treatment for people in the upper half of the risk distribution. Equity can also be improved for those who are intolerant to statins by treating them to the same target as those who can tolerate statins.

## Conclusions

As part of the 2023 update of the NICE guideline on CVD, this model-based cost-effectiveness analysis sought to identify the most cost-effective cholesterol threshold for lipid therapy escalation in England in people with established CVD taking a statin. It was most cost-effective for people to have their therapy escalated if their LDL-C was ≥ 2.2 mmol/L (or equivalent non-HDL-C of 2.9 mmol/L) at NICE’s lower cost per QALY of £20,000. However, 2.0 mmol/L LDL-C (or equivalent 2.6 mmol/L non-HDL-C) produced more health gain and was found to be more cost-effective at £20,000 per QALY in a significant proportion (38%) of the probabilistic simulations. Therefore, 2.0 mmol/L LDL-C (or equivalent 2.6 mmol/L non-HDL-C) was recommended by the guideline committee for the secondary prevention of CVD in England. Sensitivity analysis of the statin intolerant population did not affect the optimal value of the cholesterol threshold. This study used a flexible Markov model structure to account for population heterogeneity and combining high quality RCT data and large-scale real-world data from England. The results demonstrate the importance of establishing evidence of cost-effectiveness for cholesterol treatment escalation thresholds.

## Supplementary Information

Below is the link to the electronic supplementary material.

Supplementary file1 (DOCX 84 KB)

## Declarations

### Data and Code Availability

A version of the model with confidential data replaced with dummy data may be available by reasonable request from the corresponding author.
