# Supplementary material for: Using a Markov Model and Real-World Evidence to Identify the Most Cost-Effective Cholesterol Treatment Escalation Threshold for the Secondary Prevention of Cardiovascular Disease
Source: Appl Health Econ Health Policy. 2025 May 24;23(5):869–83. doi: 10.1007/s40258-025-00977-6 (PMC12364731; doi:10.1007/s40258-025-00977-6)
Supplement: Supplementary file 1 — Supplementary file1 (DOCX 84 KB) [file 40258_2025_977_MOESM1_ESM.docx]

Supplementary materials

**SUPPLEMENTARY INFORMATION**

**Article title:** Using a Markov model and real-world evidence to identify the most cost-effective cholesterol treatment escalation threshold for the secondary prevention of cardiovascular disease

**Journal:** Applied Health Economics and Health Policy

**Authors:** Mariani A., Mohiuddin S., Muller P., Samarasekera E., Swain S., Mills J., Patel R., Preiss D., Shantsila E., Downing B.C., Lonergan M., Rowark S., Welton N.J., Williams R., Wonderling D.

**Corresponding author:** Alfredo Mariani, National Institute for Health and Care Excellence, UK

email: [alfredo.mariani@nice.org.uk](mailto:alfredo.mariani@nice.org.uk)

CPRD analysis

Table S1: Admission rates by age and type of admission – men*

|  | Person-years | Admissions | Rate | Rate - Lower 95% CL | Rate – upper 95% CL |
| --- | --- | --- | --- | --- | --- |
| **Ischaemic stroke** | | | | | |
| 18-44 | 11,990 | 60 | 0.0050 | 0.0039 | 0.0064 |
| 45-49 | 21,896 | 104 | 0.0047 | 0.0039 | 0.0058 |
| 50-54 | 45,156 | 213 | 0.0047 | 0.0041 | 0.0054 |
| 55-59 | 71,734 | 392 | 0.0055 | 0.0049 | 0.0060 |
| 60-64 | 99,160 | 628 | 0.0063 | 0.0059 | 0.0068 |
| 65-69 | 138,615 | 850 | 0.0061 | 0.0057 | 0.0066 |
| 70-74 | 154,737 | 1,270 | 0.0082 | 0.0078 | 0.0087 |
| 75-79 | 149,005 | 1,716 | 0.0115 | 0.0110 | 0.0121 |
| 80-84 | 127,111 | 1,875 | 0.0148 | 0.0141 | 0.0154 |
| 85-89 | 77,389 | 1,420 | 0.0183 | 0.0174 | 0.0193 |
| 90+ | 31,887 | 762 | 0.0239 | 0.0223 | 0.0257 |
| **Myocardial infarction** | | | | | |
| 18-44 | 11,990 | 142 | 0.0118 | 0.0100 | 0.0140 |
| 45-49 | 21,896 | 230 | 0.0105 | 0.0092 | 0.0120 |
| 50-54 | 45,156 | 531 | 0.0118 | 0.0108 | 0.0128 |
| 55-59 | 71,734 | 840 | 0.0117 | 0.0109 | 0.0125 |
| 60-64 | 99,160 | 1,104 | 0.0111 | 0.0105 | 0.0118 |
| 65-69 | 138,615 | 1,409 | 0.0102 | 0.0096 | 0.0107 |
| 70-74 | 154,737 | 1,849 | 0.0119 | 0.0114 | 0.0125 |
| 75-79 | 149,005 | 2,040 | 0.0137 | 0.0131 | 0.0143 |
| 80-84 | 127,111 | 2,162 | 0.0170 | 0.0163 | 0.0177 |
| 85-89 | 77,389 | 1,638 | 0.0212 | 0.0202 | 0.0222 |
| 90+ | 31,887 | 916 | 0.0287 | 0.0269 | 0.0306 |
| **Unstable angina** | | | | | |
| 18-44 | 11,990 | 106 | 0.0088 | 0.0073 | 0.0107 |
| 45-49 | 21,896 | 200 | 0.0091 | 0.0080 | 0.0105 |
| 50-54 | 45,156 | 384 | 0.0085 | 0.0077 | 0.0094 |
| 55-59 | 71,734 | 514 | 0.0072 | 0.0066 | 0.0078 |
| 60-64 | 99,160 | 669 | 0.0067 | 0.0063 | 0.0073 |
| 65-69 | 138,615 | 786 | 0.0057 | 0.0053 | 0.0061 |
| 70-74 | 154,737 | 756 | 0.0049 | 0.0045 | 0.0052 |
| 75-79 | 149,005 | 795 | 0.0053 | 0.0050 | 0.0057 |
| 80-84 | 127,111 | 675 | 0.0053 | 0.0049 | 0.0057 |
| 85-89 | 77,389 | 404 | 0.0052 | 0.0047 | 0.0058 |
| 90+ | 31,887 | 158 | 0.0050 | 0.0042 | 0.0058 |
| **Non-coronary revascularisation** | | | | | |
| 18-44 | 11,990 | 47 | 0.0039 | 0.0029 | 0.0052 |
| 45-49 | 21,896 | 85 | 0.0039 | 0.0031 | 0.0048 |
| 50-54 | 45,156 | 309 | 0.0068 | 0.0061 | 0.0077 |
| 55-59 | 71,734 | 556 | 0.0078 | 0.0071 | 0.0084 |
| 60-64 | 99,160 | 909 | 0.0092 | 0.0086 | 0.0098 |
| 65-69 | 138,615 | 1,333 | 0.0096 | 0.0091 | 0.0101 |
| 70-74 | 154,737 | 1,432 | 0.0093 | 0.0088 | 0.0097 |
| 75-79 | 149,005 | 1,293 | 0.0087 | 0.0082 | 0.0092 |
| 80-84 | 127,111 | 932 | 0.0073 | 0.0069 | 0.0078 |
| 85-89 | 77,389 | 431 | 0.0056 | 0.0051 | 0.0061 |
| 90+ | 31,887 | 122 | 0.0038 | 0.0032 | 0.0046 |
| **Elective coronary revascularisation** | | | | | |
| 18-44 | 11,990 | 264 | 0.0220 | 0.0195 | 0.0248 |
| 45-49 | 21,896 | 590 | 0.0269 | 0.0249 | 0.0292 |
| 50-54 | 45,156 | 1,182 | 0.0262 | 0.0247 | 0.0277 |
| 55-59 | 71,734 | 1,736 | 0.0242 | 0.0231 | 0.0254 |
| 60-64 | 99,160 | 2,051 | 0.0207 | 0.0198 | 0.0216 |
| 65-69 | 138,615 | 2,187 | 0.0158 | 0.0151 | 0.0165 |
| 70-74 | 154,737 | 2,102 | 0.0136 | 0.0130 | 0.0142 |
| 75-79 | 149,005 | 1,677 | 0.0113 | 0.0107 | 0.0118 |
| 80-84 | 127,111 | 873 | 0.0069 | 0.0064 | 0.0073 |
| 85-89 | 77,389 | 276 | 0.0036 | 0.0032 | 0.0040 |
| 90+ | 31,887 | 33 | 0.0010 | 0.0007 | 0.0015 |

* Men with CVD on a statin but not on other lipid lowering therapy

Table S2: Admission rates by age and type of admission – women*

| Person-years | Admissions | Rate | Rate - Lower 95% CL | Rate – upper 95% CL |
| --- | --- | --- | --- | --- |
| **Ischaemic stroke** | | | | |
| 5,002 | 50 | 0.0100 | 0.0076 | 0.0132 |
| 8,668 | 82 | 0.0095 | 0.0076 | 0.0117 |
| 17,398 | 109 | 0.0063 | 0.0052 | 0.0076 |
| 27,425 | 171 | 0.0062 | 0.0054 | 0.0072 |
| 40,018 | 294 | 0.0073 | 0.0066 | 0.0082 |
| 60,693 | 408 | 0.0067 | 0.0061 | 0.0074 |
| 79,618 | 774 | 0.0097 | 0.0091 | 0.0104 |
| 92,582 | 1,265 | 0.0137 | 0.0129 | 0.0144 |
| 98,470 | 1,734 | 0.0176 | 0.0168 | 0.0185 |
| 78,778 | 1,856 | 0.0236 | 0.0225 | 0.0247 |
| 50,480 | 1,522 | 0.0302 | 0.0287 | 0.0317 |
| **Myocardial infarction** | | | | |
| 5,002 | 42 | 0.0084 | 0.0062 | 0.0114 |
| 8,668 | 79 | 0.0091 | 0.0073 | 0.0114 |
| 17,398 | 159 | 0.0091 | 0.0078 | 0.0107 |
| 27,425 | 191 | 0.0070 | 0.0060 | 0.0080 |
| 40,018 | 314 | 0.0078 | 0.0070 | 0.0088 |
| 60,693 | 473 | 0.0078 | 0.0071 | 0.0085 |
| 79,618 | 659 | 0.0083 | 0.0077 | 0.0089 |
| 92,582 | 1,044 | 0.0113 | 0.0106 | 0.0120 |
| 98,470 | 1,336 | 0.0136 | 0.0129 | 0.0143 |
| 78,778 | 1,302 | 0.0165 | 0.0157 | 0.0175 |
| 50,480 | 992 | 0.0197 | 0.0185 | 0.0209 |
| **Unstable angina** | | | | |
| 5,002 | 56 | 0.0113 | 0.0087 | 0.0146 |
| 8,668 | 89 | 0.0102 | 0.0083 | 0.0126 |
| 17,398 | 152 | 0.0087 | 0.0074 | 0.0102 |
| 27,425 | 214 | 0.0078 | 0.0068 | 0.0089 |
| 40,018 | 254 | 0.0063 | 0.0056 | 0.0072 |
| 60,693 | 331 | 0.0055 | 0.0049 | 0.0061 |
| 79,618 | 428 | 0.0054 | 0.0049 | 0.0059 |
| 92,582 | 460 | 0.0050 | 0.0045 | 0.0054 |
| 98,470 | 499 | 0.0051 | 0.0046 | 0.0055 |
| 78,778 | 354 | 0.0045 | 0.0040 | 0.0050 |
| 50,480 | 214 | 0.0042 | 0.0037 | 0.0048 |
| **Non-coronary revascularisation** | | | | |
| 5,002 | 23 | 0.0046 | 0.0031 | 0.0069 |
| 8,668 | 56 | 0.0065 | 0.0050 | 0.0084 |
| 17,398 | 130 | 0.0075 | 0.0063 | 0.0089 |
| 27,425 | 191 | 0.0070 | 0.0060 | 0.0080 |
| 40,018 | 269 | 0.0067 | 0.0060 | 0.0076 |
| 60,693 | 365 | 0.0060 | 0.0054 | 0.0067 |
| 79,618 | 484 | 0.0061 | 0.0056 | 0.0066 |
| 92,582 | 648 | 0.0070 | 0.0065 | 0.0076 |
| 98,470 | 524 | 0.0053 | 0.0049 | 0.0058 |
| 78,778 | 323 | 0.0041 | 0.0037 | 0.0046 |
| 50,480 | 136 | 0.0027 | 0.0023 | 0.0032 |
| **Elective coronary revascularisation** | | | | |
| 5,002 | 38 | 0.0076 | 0.0055 | 0.0104 |
| 8,668 | 103 | 0.0119 | 0.0098 | 0.0144 |
| 17,398 | 216 | 0.0124 | 0.0109 | 0.0142 |
| 27,425 | 324 | 0.0118 | 0.0106 | 0.0132 |
| 40,018 | 423 | 0.0106 | 0.0096 | 0.0116 |
| 60,693 | 557 | 0.0092 | 0.0084 | 0.0100 |
| 79,618 | 617 | 0.0077 | 0.0072 | 0.0084 |
| 92,582 | 595 | 0.0064 | 0.0059 | 0.0070 |
| 98,470 | 353 | 0.0036 | 0.0032 | 0.0040 |
| 78,778 | 123 | 0.0016 | 0.0013 | 0.0019 |
| 50,480 | 24 | 0.0005 | 0.0003 | 0.0007 |

* Women with CVD on a statin but not on other lipid lowering therapy

Table S3: Mortality rates by age and type of event in last 12 months – men*

|  | Person-years | NCV deaths | CV deaths | All deaths | Rate | Rate - Lower 95% CL | Rate – upper 95% CL |
| --- | --- | --- | --- | --- | --- | --- | --- |
| **Ischaemic stroke** | | | | | | | |
| 18-44 | 121 | 1 | 1 | 2 | 0.0165 | 0.0041 | 0.0659 |
| 45-49 | 155 | 1 | 7 | 8 | 0.0517 | 0.0258 | 0.1033 |
| 50-54 | 303 | 11 | 6 | 17 | 0.0560 | 0.0348 | 0.0901 |
| 55-59 | 412 | 11 | 24 | 35 | 0.0850 | 0.0610 | 0.1184 |
| 60-64 | 610 | 27 | 27 | 54 | 0.0886 | 0.0678 | 0.1156 |
| 65-69 | 783 | 66 | 60 | 126 | 0.1608 | 0.1351 | 0.1915 |
| 70-74 | 1,067 | 99 | 77 | 176 | 0.1650 | 0.1423 | 0.1912 |
| 75-79 | 1,246 | 172 | 147 | 319 | 0.2559 | 0.2293 | 0.2856 |
| 80-84 | 1,294 | 241 | 268 | 509 | 0.3935 | 0.3607 | 0.4292 |
| 85-89 | 883 | 216 | 295 | 511 | 0.5784 | 0.5303 | 0.6308 |
| 90+ | 418 | 154 | 208 | 362 | 0.8653 | 0.7806 | 0.9591 |
| **Myocardial infarction** | | | | | | | |
| 18-44 | 310 | 3 | 0 | 3 | 0.0097 | 0.0031 | 0.0300 |
| 45-49 | 426 | 8 | 3 | 11 | 0.0258 | 0.0143 | 0.0466 |
| 50-54 | 771 | 15 | 12 | 27 | 0.0350 | 0.0240 | 0.0510 |
| 55-59 | 1,058 | 27 | 11 | 38 | 0.0359 | 0.0261 | 0.0494 |
| 60-64 | 1,194 | 64 | 17 | 81 | 0.0678 | 0.0545 | 0.0843 |
| 65-69 | 1,419 | 128 | 34 | 162 | 0.1141 | 0.0979 | 0.1332 |
| 70-74 | 1,532 | 221 | 60 | 281 | 0.1834 | 0.1631 | 0.2061 |
| 75-79 | 1,518 | 309 | 91 | 400 | 0.2635 | 0.2389 | 0.2906 |
| 80-84 | 1,494 | 434 | 128 | 562 | 0.3763 | 0.3464 | 0.4087 |
| 85-89 | 1,020 | 433 | 153 | 586 | 0.5743 | 0.5296 | 0.6227 |
| 90+ | 519 | 351 | 104 | 455 | 0.8768 | 0.7999 | 0.9612 |
| **Unstable angina** | | | | | | | |
| 18-44 | 227 | 0 | 1 | 1 | 0.0044 | 0.0006 | 0.0312 |
| 45-49 | 416 | 1 | 2 | 3 | 0.0072 | 0.0023 | 0.0224 |
| 50-54 | 768 | 3 | 3 | 6 | 0.0078 | 0.0035 | 0.0174 |
| 55-59 | 1,074 | 15 | 4 | 19 | 0.0177 | 0.0113 | 0.0277 |
| 60-64 | 1,257 | 23 | 5 | 28 | 0.0223 | 0.0154 | 0.0322 |
| 65-69 | 1,502 | 33 | 8 | 41 | 0.0273 | 0.0201 | 0.0371 |
| 70-74 | 1,499 | 55 | 23 | 78 | 0.0520 | 0.0417 | 0.0650 |
| 75-79 | 1,567 | 91 | 33 | 124 | 0.0791 | 0.0663 | 0.0943 |
| 80-84 | 1,361 | 123 | 44 | 167 | 0.1227 | 0.1054 | 0.1428 |
| 85-89 | 827 | 116 | 50 | 166 | 0.2008 | 0.1725 | 0.2338 |
| 90+ | 327 | 73 | 27 | 100 | 0.3060 | 0.2515 | 0.3722 |
| **Non-coronary revascularisation** | | | | | | | |
| 18-44 | 37 | 0 | 1 | 1 | 0.0268 | 0.0038 | 0.1900 |
| 45-49 | 74 | 3 | 0 | 3 | 0.0405 | 0.0131 | 0.1256 |
| 50-54 | 235 | 7 | 3 | 10 | 0.0426 | 0.0229 | 0.0791 |
| 55-59 | 460 | 9 | 8 | 17 | 0.0370 | 0.0230 | 0.0595 |
| 60-64 | 710 | 21 | 7 | 28 | 0.0394 | 0.0272 | 0.0571 |
| 65-69 | 1,003 | 64 | 20 | 84 | 0.0837 | 0.0676 | 0.1037 |
| 70-74 | 1,078 | 102 | 33 | 135 | 0.1253 | 0.1058 | 0.1483 |
| 75-79 | 935 | 114 | 38 | 152 | 0.1626 | 0.1387 | 0.1906 |
| 80-84 | 692 | 104 | 35 | 139 | 0.2008 | 0.1701 | 0.2372 |
| 85-89 | 312 | 94 | 24 | 118 | 0.3788 | 0.3163 | 0.4537 |
| 90+ | 90 | 32 | 7 | 39 | 0.4326 | 0.3161 | 0.5921 |
| **Elective coronary revascularisation** | | | | | | | |
| 18-44 | 251 | 0 | 0 | 0 | 0.0000 |  |  |
| 45-49 | 537 | 0 | 2 | 2 | 0.0037 | 0.0009 | 0.0149 |
| 50-54 | 1,104 | 3 | 2 | 5 | 0.0045 | 0.0019 | 0.0109 |
| 55-59 | 1,610 | 3 | 5 | 8 | 0.0050 | 0.0025 | 0.0099 |
| 60-64 | 1,928 | 6 | 8 | 14 | 0.0073 | 0.0043 | 0.0123 |
| 65-69 | 2,020 | 16 | 12 | 28 | 0.0139 | 0.0096 | 0.0201 |
| 70-74 | 1,974 | 38 | 12 | 50 | 0.0253 | 0.0192 | 0.0334 |
| 75-79 | 1,561 | 44 | 31 | 75 | 0.0480 | 0.0383 | 0.0602 |
| 80-84 | 825 | 43 | 13 | 56 | 0.0679 | 0.0522 | 0.0882 |
| 85-89 | 259 | 21 | 3 | 24 | 0.0928 | 0.0622 | 0.1385 |
| 90+ | 35 | 6 | 0 | 6 | 0.1718 | 0.0772 | 0.3823 |
| **No event in last 12 months** | | | | | | | |
| 18-44 | 6,834 | 28 | 4 | 32 | 0.0046 | 0.0033 | 0.0066 |
| 45-49 | 13,613 | 49 | 21 | 70 | 0.0051 | 0.0041 | 0.0065 |
| 50-54 | 29,894 | 176 | 55 | 231 | 0.0077 | 0.0068 | 0.0088 |
| 55-59 | 49,439 | 410 | 114 | 523 | 0.0106 | 0.0097 | 0.0115 |
| 60-64 | 69,732 | 859 | 222 | 1,081 | 0.0155 | 0.0146 | 0.0165 |
| 65-69 | 99,029 | 1,603 | 517 | 2,120 | 0.0214 | 0.0205 | 0.0223 |
| 70-74 | 110,185 | 2,706 | 706 | 3,411 | 0.0310 | 0.0299 | 0.0320 |
| 75-79 | 105,509 | 3,958 | 1,034 | 4,992 | 0.0473 | 0.0460 | 0.0486 |
| 80-84 | 89,281 | 5,285 | 1,382 | 6,668 | 0.0747 | 0.0729 | 0.0765 |
| 85-89 | 53,852 | 5,269 | 1,374 | 6,643 | 0.1234 | 0.1204 | 0.1264 |
| 90+ | 21,968 | 3,759 | 1,003 | 4,763 | 0.2168 | 0.2107 | 0.2230 |
| **All** | | | | | | | |
| 18-44 | 7,780 | 32 | 7 | 39 | 0.0050 | 0.0036 | 0.0068 |
| 45-49 | 15,221 | 62 | 35 | 97 | 0.0064 | 0.0052 | 0.0078 |
| 50-54 | 33,076 | 215 | 81 | 296 | 0.0089 | 0.0080 | 0.0100 |
| 55-59 | 54,053 | 475 | 166 | 640 | 0.0118 | 0.0110 | 0.0128 |
| 60-64 | 75,432 | 1,000 | 286 | 1,286 | 0.0170 | 0.0161 | 0.0180 |
| 65-69 | 105,756 | 1,910 | 651 | 2,561 | 0.0242 | 0.0233 | 0.0252 |
| 70-74 | 117,334 | 3,221 | 911 | 4,131 | 0.0352 | 0.0342 | 0.0363 |
| 75-79 | 112,337 | 4,688 | 1,374 | 6,062 | 0.0540 | 0.0526 | 0.0553 |
| 80-84 | 94,946 | 6,230 | 1,870 | 8,101 | 0.0853 | 0.0835 | 0.0872 |
| 85-89 | 57,153 | 6,149 | 1,899 | 8,048 | 0.1408 | 0.1378 | 0.1439 |
| 90+ | 23,358 | 4,375 | 1,349 | 5,725 | 0.2451 | 0.2388 | 0.2515 |
| All | 696,446 | 28,356 | 8,630 | 36,986 |  |  |  |

* Men with CVD on a statin but not on other lipid lowering therapy

CV= modifiable cardiovascular deaths; NCV=all other deaths

Table S4: Mortality rates by age and type of event in last 12 months – women*

|  | Person-years | NCV deaths | CV deaths | All deaths | Rate | Rate - Lower 95% CL | Rate – upper 95% CL |
| --- | --- | --- | --- | --- | --- | --- | --- |
| **Ischaemic stroke** | | | | | | | |
| 18-44 | 101 | 2 | 0 | 2 | 0.0199 | 0.0050 | 0.0794 |
| 45-49 | 105 | 3 | 1 | 4 | 0.0382 | 0.0143 | 0.1017 |
| 50-54 | 158 | 4 | 3 | 7 | 0.0442 | 0.0211 | 0.0927 |
| 55-59 | 198 | 8 | 3 | 11 | 0.0556 | 0.0308 | 0.1004 |
| 60-64 | 286 | 22 | 11 | 33 | 0.1152 | 0.0819 | 0.1621 |
| 65-69 | 394 | 46 | 33 | 79 | 0.2004 | 0.1607 | 0.2498 |
| 70-74 | 625 | 66 | 63 | 129 | 0.2064 | 0.1737 | 0.2452 |
| 75-79 | 926 | 126 | 134 | 260 | 0.2809 | 0.2488 | 0.3172 |
| 80-84 | 1,195 | 222 | 258 | 480 | 0.4015 | 0.3672 | 0.4391 |
| 85-89 | 1,143 | 287 | 366 | 653 | 0.5714 | 0.5292 | 0.6169 |
| 90+ | 745 | 315 | 468 | 783 | 1.0514 | 0.9803 | 1.1277 |
| **Myocardial infarction** | | | | | | | |
| 18-44 | 91 | 1 | 1 | 2 | 0.0220 | 0.0055 | 0.0881 |
| 45-49 | 124 | 3 | 2 | 5 | 0.0404 | 0.0168 | 0.0971 |
| 50-54 | 219 | 9 | 2 | 11 | 0.0503 | 0.0278 | 0.0908 |
| 55-59 | 279 | 17 | 1 | 18 | 0.0645 | 0.0406 | 0.1024 |
| 60-64 | 356 | 27 | 10 | 37 | 0.1039 | 0.0753 | 0.1434 |
| 65-69 | 494 | 59 | 12 | 71 | 0.1436 | 0.1138 | 0.1812 |
| 70-74 | 621 | 85 | 19 | 104 | 0.1674 | 0.1381 | 0.2028 |
| 75-79 | 822 | 141 | 51 | 192 | 0.2337 | 0.2029 | 0.2692 |
| 80-84 | 958 | 295 | 65 | 360 | 0.3758 | 0.3389 | 0.4167 |
| 85-89 | 879 | 320 | 88 | 408 | 0.4642 | 0.4213 | 0.5115 |
| 90+ | 615 | 355 | 89 | 444 | 0.7215 | 0.6574 | 0.7918 |
| **Unstable angina** | | | | | | | |
| 18-44 | 127 | 1 | 0 | 1 | 0.0079 | 0.0011 | 0.0557 |
| 45-49 | 200 | 1 | 1 | 2 | 0.0100 | 0.0025 | 0.0399 |
| 50-54 | 355 | 1 | 0 | 1 | 0.0028 | 0.0004 | 0.0200 |
| 55-59 | 484 | 4 | 3 | 7 | 0.0145 | 0.0069 | 0.0303 |
| 60-64 | 586 | 2 | 4 | 6 | 0.0102 | 0.0046 | 0.0228 |
| 65-69 | 704 | 16 | 5 | 21 | 0.0298 | 0.0195 | 0.0458 |
| 70-74 | 955 | 29 | 6 | 35 | 0.0366 | 0.0263 | 0.0510 |
| 75-79 | 993 | 46 | 13 | 59 | 0.0594 | 0.0460 | 0.0767 |
| 80-84 | 1,062 | 76 | 28 | 104 | 0.0979 | 0.0808 | 0.1187 |
| 85-89 | 774 | 75 | 27 | 102 | 0.1318 | 0.1086 | 0.1600 |
| 90+ | 459 | 78 | 30 | 108 | 0.2353 | 0.1948 | 0.2841 |
| **Non-coronary revascularisation** | | | | | | | |
| 18-44 | 19 | 1 | 0 | 1 | 0.0522 | 0.0074 | 0.3708 |
| 45-49 | 40 | 2 | 0 | 2 | 0.0495 | 0.0124 | 0.1979 |
| 50-54 | 91 | 1 | 0 | 1 | 0.0110 | 0.0016 | 0.0781 |
| 55-59 | 163 | 8 | 1 | 9 | 0.0552 | 0.0287 | 0.1061 |
| 60-64 | 205 | 8 | 5 | 13 | 0.0633 | 0.0368 | 0.1090 |
| 65-69 | 288 | 27 | 2 | 29 | 0.1005 | 0.0699 | 0.1447 |
| 70-74 | 370 | 28 | 4 | 32 | 0.0866 | 0.0612 | 0.1224 |
| 75-79 | 478 | 58 | 10 | 68 | 0.1423 | 0.1122 | 0.1804 |
| 80-84 | 402 | 54 | 20 | 74 | 0.1840 | 0.1465 | 0.2311 |
| 85-89 | 234 | 48 | 6 | 54 | 0.2308 | 0.1768 | 0.3014 |
| 90+ | 113 | 36 | 7 | 43 | 0.3801 | 0.2819 | 0.5125 |
| **Elective coronary revascularisation** | | | | | | | |
| 18-44 | 37 | 0 | 0 | 0 | 0.0000 |  |  |
| 45-49 | 88 | 0 | 1 | 1 | 0.0114 | 0.0016 | 0.0807 |
| 50-54 | 206 | 2 | 0 | 2 | 0.0097 | 0.0024 | 0.0389 |
| 55-59 | 299 | 2 | 2 | 4 | 0.0134 | 0.0050 | 0.0357 |
| 60-64 | 396 | 11 | 3 | 14 | 0.0353 | 0.0209 | 0.0596 |
| 65-69 | 528 | 5 | 2 | 7 | 0.0133 | 0.0063 | 0.0278 |
| 70-74 | 572 | 12 | 4 | 16 | 0.0280 | 0.0171 | 0.0456 |
| 75-79 | 557 | 14 | 4 | 18 | 0.0323 | 0.0204 | 0.0513 |
| 80-84 | 329 | 4 | 5 | 9 | 0.0273 | 0.0142 | 0.0525 |
| 85-89 | 126 | 2 | 6 | 8 | 0.0637 | 0.0318 | 0.1273 |
| 90+ | 26 | 1 | 0 | 1 | 0.0385 | 0.0054 | 0.2730 |
| **No event in last 12 months** | | | | | | | |
| 18-44 | 3,116 | 21 | 2 | 23 | 0.0073 | 0.0048 | 0.0110 |
| 45-49 | 5,629 | 30 | 13 | 42 | 0.0075 | 0.0056 | 0.0102 |
| 50-54 | 11,807 | 98 | 12 | 110 | 0.0093 | 0.0077 | 0.0112 |
| 55-59 | 19,068 | 181 | 27 | 208 | 0.0109 | 0.0095 | 0.0125 |
| 60-64 | 27,350 | 376 | 63 | 439 | 0.0160 | 0.0146 | 0.0176 |
| 65-69 | 41,519 | 761 | 126 | 888 | 0.0214 | 0.0200 | 0.0228 |
| 70-74 | 54,545 | 1,328 | 226 | 1,554 | 0.0285 | 0.0271 | 0.0299 |
| 75-79 | 62,893 | 2,196 | 400 | 2,596 | 0.0413 | 0.0397 | 0.0429 |
| 80-84 | 66,930 | 3,498 | 817 | 4,315 | 0.0645 | 0.0626 | 0.0664 |
| 85-89 | 53,186 | 4,531 | 1,007 | 5,539 | 0.1041 | 0.1014 | 0.1069 |
| 90+ | 34,057 | 5,412 | 1,301 | 6,714 | 0.1971 | 0.1925 | 0.2019 |
| **All** | | | | | | | |
| 18-44 | 3,491 | 26 | 3 | 29 | 0.0082 | 0.0057 | 0.0118 |
| 45-49 | 6,186 | 39 | 18 | 56 | 0.0091 | 0.0070 | 0.0118 |
| 50-54 | 12,835 | 115 | 17 | 132 | 0.0103 | 0.0086 | 0.0122 |
| 55-59 | 20,491 | 220 | 37 | 257 | 0.0125 | 0.0111 | 0.0141 |
| 60-64 | 29,181 | 446 | 96 | 542 | 0.0186 | 0.0171 | 0.0202 |
| 65-69 | 43,928 | 914 | 180 | 1,095 | 0.0249 | 0.0235 | 0.0264 |
| 70-74 | 57,689 | 1,548 | 322 | 1,870 | 0.0324 | 0.0310 | 0.0339 |
| 75-79 | 66,668 | 2,581 | 612 | 3,193 | 0.0479 | 0.0463 | 0.0496 |
| 80-84 | 70,877 | 4,149 | 1,193 | 5,342 | 0.0754 | 0.0734 | 0.0774 |
| 85-89 | 56,341 | 5,263 | 1,500 | 6,764 | 0.1201 | 0.1172 | 0.1229 |
| 90+ | 36,015 | 6,197 | 1,895 | 8,093 | 0.2247 | 0.2199 | 0.2297 |
| All | 403,701 | 21,497 | 5,874 | 27,371 |  |  |  |

* Women with CVD on a statin but not on other lipid lowering therapy

CV= modifiable cardiovascular deaths; NCV=all other deaths

# Non-HDL-C analysis

## Relative treatment effect

The only CTT collaboration estimate of relative risk reduction in non-HDL-C was for MI and CHD death, which was 0.79, or a 21% reduction per 1 mmol/litre reduction[1]. The corresponding figure for LDL-C in the same CTT publication was 26% reduction. The non-HDL-C reduction specifically for MI was estimated as the 21% multiplied by the LDL-C reduction for MI divided by the LDL-C reduction for MI and stroke (= 21% X (27%/26%). The other non-HDL treatment effects were approximated in the same manner (see Table 5).

Table S5: Relative effect on vascular events and mortality per 1 mmol/litre reduction in non-HDL-C

| Event | Application in model base case | Relative risk reduction | Source |
| --- | --- | --- | --- |
| Major CVD event | Non-coronary revascularisation | 0.81 | Derived using the non-HDL-C effect for MI and stroke combined with ratio of relevant LDL-C effects(2017; Cholesterol Treatment Trialists, et al., 2010) |
| Any coronary revascularisation | Any coronary revascularisation | 0.78 | Derived using the non-HDL-C effect for MI and stroke combined with ratio of relevant LDL-C effects(2017; Cholesterol Treatment Trialists, Baigent, Blackwell, Emberson, Holland, Reith, Bhala, Peto, Barnes, Keech, Simes and Collins, 2010) |
| Ischaemic stroke | Ischaemic stroke | 0.81 | Derived using the non-HDL-C effect for MI and stroke combined with ratio of relevant LDL-C effects(2017; Cholesterol Treatment Trialists, Baigent, Blackwell, Emberson, Holland, Reith, Bhala, Peto, Barnes, Keech, Simes and Collins, 2010) |
| Myocardial infarction | Myocardial infarction | 0.77 | Derived using the non-HDL-C effect for MI and stroke combined with ratio of relevant LDL-C effects(2017; Cholesterol Treatment Trialists, Baigent, Blackwell, Emberson, Holland, Reith, Bhala, Peto, Barnes, Keech, Simes and Collins, 2010) |
| CHD death | CVD death (sensitivity analysis only) | 0.83 | Derived using the non-HDL-C effect for MI and stroke combined with ratio of relevant LDL-C effects(2017; Cholesterol Treatment Trialists, Baigent, Blackwell, Emberson, Holland, Reith, Bhala, Peto, Barnes, Keech, Simes and Collins, 2010) |
| All-cause mortality | All deaths | 0.91 | Derived using the non-HDL-C effect for MI and stroke combined with ratio of relevant LDL-C effects(2017; Cholesterol Treatment Trialists, et al., 2012) |

## Non-HDL-C results

Table S6: Deterministic results of non-HDL-C analysis

|  | Optimal threshold non-HDL-C (mmol/L) | Mean QALYs | % on ezetimibe | % on inclisiran |
| --- | --- | --- | --- | --- |
| Base-case | 2.9 | 6.006 | 30.7% | 13.1% |
| £30,000 per QALY | 2.2 | 6.115 | 66.1% | 42.9% |
| CVD mortality RR | 3.7 | 5.946 | 8.0% | 3.2% |
| Only PCSK9i | 4.2 | 5.922 | 3.2% | 1.6% |
| Atorvastatin cholesterol distribution | 3.1 | 7.660 | 16.7% | 7.9% |
| Exclude unstable angina | 2.9 | 6.018 | 30.7% | 13.1% |
| Exclude TIA | 2.9 | 6.006 | 30.7% | 13.1% |
| Previous statin model Utilities | 2.9 | 5.761 | 30.7% | 13.1% |
| Inclisiran TA Utilities | 2.9 | 6.263 | 30.7% | 13.1% |
| Ezetimibe 80% adherence | 3.2 | 5.968 | 13.4% | 8.0% |
| Ezetimibe 50% adherence | 3.5 | 5.957 | 6.5% | 8.0% |
| Ezetimibe 0% adherence | 4.3 | 5.928 | 0% | 3.2% |
| Injectables 80% adherence | 2.8 | 6.006 | 33.7% | 13.4% |
| Injectables 50% adherence | 2.5 | 6.015 | 50.6% | 13.5% |
| Injectables 0% adherence | 1.3 | 5.997 | 99.6% | 0% |
| Different CVD event costs | 2.9 | 6.006 | 30.7% | 13.1% |
| Volume discounted inclisiran price | 2.7 | 6.034 | 39.5% | 20.2% |
| Higher inclisiran escalation cost | 2.9 | 6.006 | 30.7% | 13.1% |
| Pharmacist fee with ezetimibe | 2.9 | 6.006 | 30.7% | 13.1% |

Note: costs are not reported in the table due to the confidentiality of the price of inclisiran and the PCSK9 inhibitors

Single threshold stratified by sex

Table S7 and S8 show the single threshold analysis stratified by sex. The threshold was found to be higher for women, 2.3 mmol/L, and lower for men, 1.9 mmol?L

**Table S7** Proportion of people on medication and mean LDL-C at different thresholds – deterministic results (females)

| Threshold LDL-C (mmol/L) | Mean QALYs | % of people on ezetimibe at 1 year | % of people on inclisiran at 1 year | Mean LDL-C at 1 year (mmol/L) |
| --- | --- | --- | --- | --- |
| 4.0 | 5.140 | 2.2% | 0% | 1.99 |
| 3.2 | 5.155 | 7.4% | 2.2% | 1.93 |
| 2.8 | 5.177 | 12.4% | 5.6% | 1.86 |
| 2.7 | 5.185 | 16.1% | 5.8% | 1.84 |
| 2.6 | 5.193 | 17.0% | 7.4% | 1.81 |
| 2.4 | 5.214 | 26.0% | 10.1% | 1.74 |
| **2.3** | **5.225** | **27.4%** | **12.4%** | **1.70** |
| 2.2 | 5.240 | 35.4% | 16.1% | 1.63 |
| 2.0 | 5.272 | 46.7% | 22.2% | 1.52 |
| 1.9 | 5.290 | 55.0% | 27.4% | 1.44 |
| 1.8 | 5.309 | 55.0% | 35.4% | 1.36 |
| 1.7 | 5.329 | 65.5% | 37.1% | 1.31 |
| 0.5 | 5.425 | 99.7% | 99.7% | 0.81 |

Note: costs are not reported due to the confidentiality agreement on the price of inclisiran

**Table S8** Proportion of people on medication and mean LDL-C at different thresholds – deterministic results (males)

| Threshold LDL-C (mmol/L) | Mean QALYs | % of people on ezetimibe at 1 year | % of people on inclisiran at 1 year | Mean LDL-C at 1 year (mmol/L) |
| --- | --- | --- | --- | --- |
| 4.0 | 6.446 | 1.1% | 0.5% | 1.86 |
| 3.8 | 6.446 | 1.1% | 0.5% | 1.86 |
| 2.7 | 6.475 | 11.4% | 3.3% | 1.76 |
| 2.5 | 6.492 | 16.8% | 4.7% | 1.72 |
| 2.4 | 6.501 | 20.2% | 6.4% | 1.68 |
| 2.2 | 6.526 | 28.8% | 11.4% | 1.59 |
| 2.0 | 6.556 | 39.7% | 16.8% | 1.49 |
| **1.9** | **6.573** | **73.5%** | **21.4%** | **1.42** |
| 1.8 | 6.594 | 47.9% | 28.8% | 1.35 |
| 1.5 | 6.656 | 71.9% | 47.9% | 1.12 |
| 0.8 | 6.747 | 99.6% | 94.2% | 1.36 |

Note: costs are not reported due to the confidentiality agreement on the price of inclisiran

References

1. The HPS3/TIMI55–REVEAL Collaborative Group. Effects of Anacetrapib in Patients with Atherosclerotic Vascular Disease New England Journal of Medicine. 2017; 377 (13): 1217-1227.
